# AKT, a Key Transmitter of HIF-1α and AR Signaling Pathways, Has a Critical Role in the Apigetrin-Mediated Anti-Cancer Effects in Prostate Cancer Cells

**DOI:** 10.3390/biomedicines10061370

**Published:** 2022-06-09

**Authors:** You-Kyung Lee, Jung-Eun Kim, Yinzhu Xu, Hengmin Han, Jae-Hyeon Lee, Hyo-Jeong Lee

**Affiliations:** 1 Department of Cancer Preventive Material Development, Graduate School, College of Korean Medicine, Kyung Hee University, 26, Kyungheedae-ro, Dondaemun-gu, Seoul 02447, Korea; dbrud9575@naver.com (Y.-K.L.); helmin0730@khu.ac.kr (H.H.); livercaring@naver.com (J.-H.L.); 2 Department of Science in Korean Medicine, Graduate School, College of Korean Medicine, Kyung Hee University, 26, Kyungheedae-ro, Dongdaemun-gu, Seoul 02447, Korea; kimjulie4717@khu.ac.kr (J.-E.K.); xyz3402@khu.ac.kr (Y.X.)

**Keywords:** apigetrin, prostate cancer, castration-resistant prostate cancer, AKT, hypoxia, HIF-1α

## Abstract

Apigetrin is a flavonoid glycoside phytochemical that is derived from various herbs and exhibits several beneficial biological activities, including anti-oxidant, anti-inflammatory, anti-obesity, and anti-cancer effects. In the present study, we elucidated the anti-cancer effect and targeting mechanism of apigetrin in LNCaP and PC-3 cells through various experiments, including cell viability by CELLOMAX^TM^ Viability Assay kit, cell migration by scratch wound assays, and 2D-and 3D- cell growth assay. Apigetrin inhibited the viability, migration, proliferation, and growth of cells in long-term 2D- and 3D- cultures cell growth. A high dose of apigetrin induced apoptosis, as evidenced by increased cleavage of poly ADP-ribose polymerase (PARP) and caspase-3 (c-cas3) in both LNCaP and PC-3 cells. Furthermore, apigetrin inhibited AR, PSA, HIF-1α, and VEGF expression in LNCaP and PC-3 cells. Apigetrin also suppressed the hypoxia-induced HIF-1α expression in these cells. Furthermore, apigetrin reduced hypoxia-induced VEGF secretion in the culture medium and inhibited hypoxia-induced tube formation of HUVECs. Silencing of AKT revealed that the anti-cancer activity of apigetrin is mediated via AKT. Thus, our data suggest that apigetrin exerts anti-cancer effects by inhibiting AKT, a central key of HIF-1α and AR signaling, in early-and late-stage prostate cancer cells.

## 1. Introduction

Prostate cancer (PCa) is the second most common malignant tumor and the fifth leading cause of cancer-related deaths in men worldwide [1]. The androgen receptor (AR) signaling axis plays a key role in the pathogenesis of PCa. Binding of androgen to the AR initiates the signal of cell growth and proliferation in cancer [2,3,4]. Androgen deprivation therapy (ADT) is the primary treatment modality for PCa. However, castration-resistant prostate cancer (CRPC) often develops within two years of ADT initiation [3]. CRPC may present as a continuous increase in serum prostate-specific antigen (PSA) levels, progression of pre-existing disease, and/or the appearance of new metastases [5].

The expression of AR and its regulated genes are kept or not in CRPC. Furthermore, persistent activation of AR without androgen binding occurs in CRPC as a result of AR gene amplification, gain-of-function mutations, and generation of ligand-binding domain-truncated AR splice variants (AR-Vs) [4,6,7]. Therefore, AR targeting alone is insufficient to treat all PCas. 

Hypoxia is a common feature of locally advanced solid tumors [8], and approximately 50–60% of solid tumors have hypoxic regions [9]. In PCa, hypoxia has been found to correlate with higher Gleason scores [10,11] and has also been implicated in the emergence of CRPC cells. Hypoxic tumors favor malignant progression, which makes it difficult to predict the therapeutic effect of chemo- and radiation-therapy [12,13,14]. Hypoxia-inducible factor-1α (HIF-1α) is a transcription factor that regulates cancer growth, metabolism, cell proliferation, migration, angiogenesis, and apoptosis [15,16]. Once stabilized under hypoxic conditions, HIF-1α upregulates many genes essential for cancer development [16]. Hypoxia and HIF-1α have been implicated in increased resistance to androgen-targeted therapies and progression to CRPC. HIF-1α is overexpressed in various cancers, including prostate cancer, and is associated with increased risk and a poor diagnosis of PCa [17,18]. Therefore, HIF-1α is a crucial target in PCa therapy. 

Phosphatidylinositol 3-kinase (PI3K)/AKT/mammalian target of rapamycin (mTOR) signaling pathway is frequently activated in PCa [19]. PI3K/AKT/mTOR signaling plays an essential role in CRPC progression and drug resistance [20]. PI3K/AKT pathway regulates AR and HIF-1α [21,22].

Natural products have received considerable attention as treatment options and cancer chemopreventive agents. Flavonoids, the naturally occurring polyphenolic compounds in fruits and vegetables, have received considerable attention due to their anti-cancer, anti-inflammatory, anti-oxidant, and antibacterial properties [23,24,25].

We have studied and found bioactive compounds from Korean medicine herbs targeting HIF-1α and AR. We found that *Crataegus pinnatifida* ethanol extract regulates HIF-1α in hypoxic DU145 cells in prior research [26]. Additionally, we found bioactive compounds inhibiting HIF-1α such as a chlorogenic acid, apigenin, and apigetrin from *Crataegus pinnafida*. It is unusual for a natural compound to target both HIF-1α and AR. We discovered apigetrin regulates both HIF-1α and AR in LNCaP cells. Apigetrin, named apigenin 7-O-glucoside or cosmosin, is a flavonoid glycoside commonly found in natural sources. Apigetrin has various beneficial properties, including anti-oxidant, anti-obesity, anti-cancer, and anti-inflammatory effects [27,28,29,30]. Apigetrin has anti-cancer effects in human stomach, thyroid, and cervical cancer [29,31,32,33]. However, the anti-cancer effects of apigetrin in PCa have not been elucidated. Therefore, in this study, we evaluated the anti-cancer effects and the molecular mechanisms of apigetrin in PCa cells. We report dual targeting AR and HIF-1 α pathways as a potential strategy for treatment of PCa.

## 2. Materials and Methods

### 2.1. Cell Culture

The human normal prostate cell line RWPE-1 cells were purchased from American Type Culture Collection (ATCC, Manassas, VA, USA) and cultured in the keratinocyte serum free media kit (K-SFM kit, Invitrogen-GIBCO, Carlsbad, CA, USA) at 37 °C with 5% CO_2_. The human PCa cell lines LNCaP and PC-3 were obtained from the Korean cell line bank (KCLB, Seoul, Korea) and cultured in the RPMI-1640 medium with 10% fetal bovine serum and 1% penicillin/streptomycin (WelGene, Daegu, Korea) at 37 °C with 5% CO_2_. HUVECs were cultured in M199 medium with 20% fetal bovine serum, 5 U/mL heparin, 3ng/mL basic fibroblast growth factor (R&D Systems, Minneapolis, MN, USA), and 100 U/mL of antibiotic–antimycotic in 0.1% gelatin-coated flasks at 37 °C with 5% CO_2_. Apigetrin (Sigma-Aldrich, St. Louis, MO, USA) was dissolved in Dimethyl Sulfoxide (DMSO) and used for in vitro assays and mechanistic studies.

### 2.2. Cell Viability Assay

The cells (1 × 10^4^ cells/well) were seeded in a 96-well plate and treated with the indicated concentrations of apigetrin (0, 3.13, 6.25, 12.5, 25, 50, 100, and 200 μM) for 24 h. Cell viability was evaluated using a CELLOMAX^TM^ Viability Assay kit (Precaregene Co., Anyang, Gyeonggi-do, Korea), which is based on WST-8. The absorbance was measured at 450 nm using a microplate reader (Sunrise RC, Tecan, Mannedorf, Switzerland). Cell viability was expressed as the percentage of viable cells in the apigentrin-treated group versus that in the untreated control (0.05% DMSO).

### 2.3. Migration Assay

Cell migration and motility were evaluated using scratch wound assays, as previously described [34]. The LNCaP (5 × 10^5^ cells/mL) and PC-3 (2.5 × 10^5^ cells/mL) cells were seeded in a 6-well plate. When the cells became approximately 70% confluent, the monolayer was scratched with a 200 μL pipette tip to create a gap. The cells were then treated with apigetrin (25 μM) for 24 h. After incubation, the cells were fixed, and randomly chosen fields were photographed under a fluorescence microscope (Nikon, Tokyo, Japan). Cell migration was evaluated using the Image J software (NIH, Bethesda, MD, USA). 

### 2.4. Crystal Violet Staining and Cell Growth Assay

The LNCaP (5 × 10^4^ cells/mL) and PC-3 (1.5 × 10^4^ cells/mL) cells were seeded in a 6-well plate and treated with 0 and 25 μM of apigetrin at the same time and incubated for five days. After five days, the cells were fixed with 1% glutaraldehyde and then stained with crystal violet solution for 30 min. After washing with deionized water and drying, a 70% ethanol solution was used to elute the crystal violet, and absorbance was measured at 570 nm using a microplate reader (Tecan, Sunrise^TM^, Männedorf, Switzerland).

### 2.5. Caspase 3/7 Detection Assay

The LNCaP (1.5 × 10^5^ cells/mL) and PC-3 (5 × 10^4^ cells/mL) cells were seeded in a 48-well plate for 24 h and then treated with 0 and 50 μM of apigetrin for 48 h. After incubation, Cell Event^TM^ Caspase-3/7 Green Detection Reagent (Life Technologies, Burlington, ON, Canada) was added to each well and incubated for 30 min at 37 °C in the dark. Green fluorescence (indicative of apoptotic cell death) was measured using a fluorescence microscope (Nikon, Tokyo, Japan).

### 2.6. 3D Cell Culture

The LNCaP (1 × 10^5^ cells/mL) and PC-3 (1 × 10^5^ cells/mL) cells were seeded on Matrigel in 96-well plates and treated with 0, 25 and 50 μM of apigetrin in 96-well plates at the same time and incubated for 48 h and 72 h. The spheroids formed were photographed at 20× magnification under a microscope 48 h and 72 h. The spheroids were then measured and their growth was evaluated using a fluorescence microscope (Nikon, Tokyo, Japan) and Nikon NIS Elements BR Imaging software.

### 2.7. Western Blotting

The LNCaP and PC-3 cells were treated with 0, 25, and 50 μM apigetrin for 24 h to 48 h and were lysed in radioimmunoprecipitation assay (RIPA) buffer (1% NP-40, 150 mM NaCl, 50 mM Tris-HCl, pH 7.4, 0.25% sodium deoxycholate, 1 mM Na_3_VO_4_, 1 M EDTA, 1 mM NaF, and protease inhibitor cocktail). Proteins were quantified using the Bio-Rad DC^TM^ protein Assay Kit II (Bio-Rad, Hercules, CA, USA). Proteins were separated by 8%–12% sodium dodecyl sulfate-polyacrylamide gel electrophoresis and transferred to a nitrocellulose membrane (Amersham Pharmacia, Uppsala, Sweden) using the transfer system. The membranes were blocked to prevent non-specific protein binding. Subsequently, they were incubated overnight at 4 °C with the primary antibodies AR, cleaved-caspase 3 (Cell Signaling, Beverly, MA, USA), PSA (Dako, Santa Clara, CA, USA), HIF-1α (Arigo, Hsinchu, Taiwan), VEGF, AKT, p-AKT, PARP (Santa Cruz Biotechnology, Dallas, Texas, USA), or β-actin (Sigma-Aldrich, St. Louis, MO, USA). After overnight incubation, the membranes were washed with 1× TBS-T and incubated for 2 h at room temperature with a 1:5000 dilution of HRP-conjugated secondary antibody. The membranes were then washed with TBS-T. Proteins were detected using an ECL reagent (GE Healthcare, Little Chalfont, Buckinghamshire, UK).

### 2.8. Measurement of VEGF Production

VEGF levels in LNCaP cultured media were measured using a commercially available ELISA kit (R&D Systems, Minneapolis, MN, USA) according to the manufacturer’s protocol. Briefly, LNCaP cells were incubated in RPMI 1640 containing 5% charcoal-stripped serum and treated with apigetrin under hypoxic conditions. After 24 h of incubation, the supernatant was collected and the absorbance was measured at 450 nm using a microplate reader (Tecan, Sunrise^TM^, Männedorf, Switzerland).

### 2.9. Tube Formation Assay

Capillary-like tube formation assay in Matrigel was performed as previously described [35]. Twenty-four well plates were coated with growth factor-reduced Matrigel and incubated at 37 °C for 30 min. Cultured media obtained from apigetrin-treated LNCaP cells under normoxic or hypoxic conditions for 24 h was added to Matrigel-coated 24-well plates containing HUVECs (4 × 10^4^ cells, primary cells, Lonza). After incubating for 12 h, the cells were fixed with 4% paraformaldehyde, and randomly chosen fields were photographed using a light microscope (Nikon, Tokyo, Japan) at 100× magnification.

### 2.10. Statistical Analysis

The data are expressed as means ± standard deviation (SD) of three replicates per experiment. The data were analyzed using GraphPad Prism software. Significance was evaluated using Sigma Plot software. Statistical significance was set at *p* < 0.05.

## 3. Results

### 3.1. Apigetrin Inhibits Cell Proliferation and Migration of LNCaP and PC-3 Cells 

**Figure 1:**
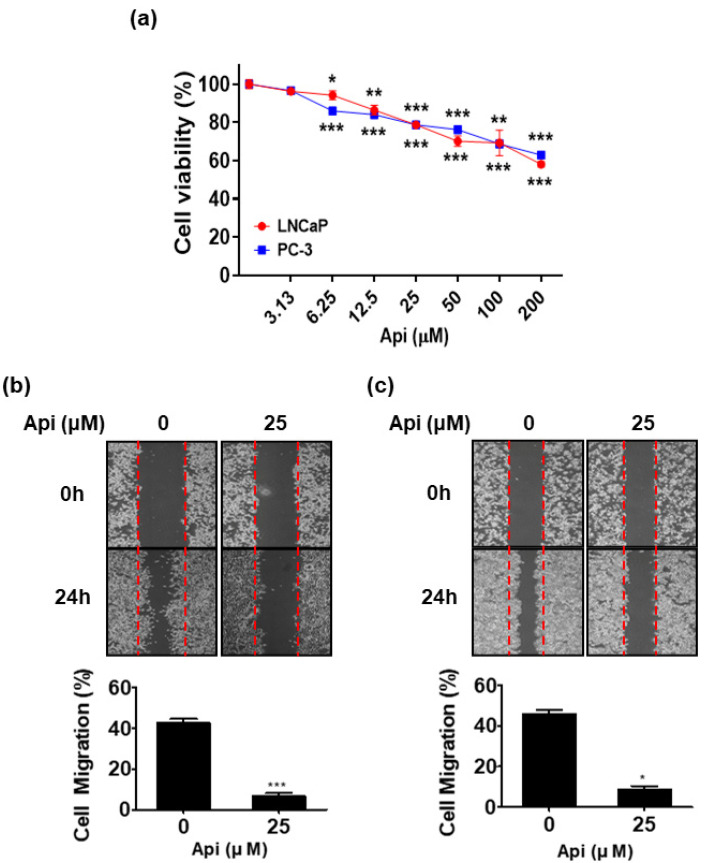
Effect of apigetrin on cell cytotoxicity and migration in LNCaP and PC-3 cells. (**a**) LNCaP and PC-3 cells were treated with the indicated concentrations of apigetrin for 24 h (n = 3). CELLOMAX^TM^ Kit based on WST-8 assay was used to measure cell viability. Results are presented as the means ± SD of three independent experiments. * *p* < 0.05, ** *p* < 0.01, and *** *p* < 0.001 versus control groups. LNCaP (**b**) and PC-3 (**c**) cells were treated with apigetrin (25 μM) for 24 h, and cell migration was assayed by wound healing assay (n = 3). The number of cells migrating into the scratched area was photographed (×100) and calculated as a migration percentage. The bar graph represents the proliferation percentage. * *p* < 0.05 and *** p < 0.001 (in compared to untreated control).

To assess the cell cytotoxicity of apigetrin, LNCaP and PC-3 human PCa cells and RWPE-1 prostate normal cells were treated with the indicated concentrations of apigetrin (0, 3.13, 6.25, 12.5, 25, 50, 100, and 200 μM) and a WST-8 assay was performed. Apigetrin demonstrated a gradual increase in toxicity in LNCaP and PC-3 cells in a concentration-dependent manner (Figure 1a). Interestingly, the longer the duration of exposure of LNCaP and PC-3 cells to apigetrin, the greater the cytotoxicity induced by apigetrin. Apigetrin (50 μM) showed a significant difference in cytotoxicity between 24 h and 48 h in LNCaP and PC-3 cells. Apigetrin (50 μM) increased cytotoxicity by 24% at 24 h and 51% at 48 h in LNCaP cells. Similarly, apigetrin (50 μM) increased cytotoxicity by 30% at 24 h and 61% at 48 h (data not shown) in PC-3 cells. As shown in Figure 1b,c, apigetrin significantly decreased cell migration by 26.7% and 11.3% in LNCaP and PC-3 cells, respectively, compared with the untreated controls. 

### 3.2. Apigetrin Enhances Inhibition of Cell Growth in Long-Term Culture 

**Figure 2:**
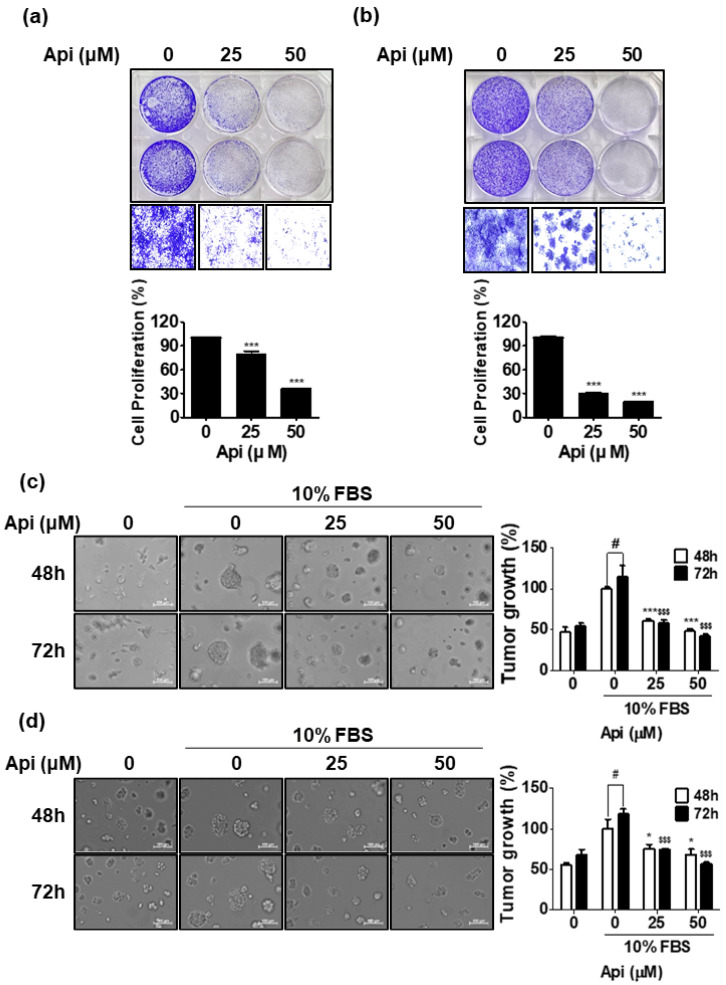
Effect of apigetrin on the growth of LNCaP and PC-3 cells in 2D and 3D cultures. LNCaP (**a**) and PC-3 (**b**) cells were treated with apigetrin (25 and 50 μM) and maintained for 5 days. The cells were resolved in 70% ethanol after washing with distilled water, and crystal violet absorbance was read using a microplate reader. Data represent the mean ± SD of two independent experiments. *** *p* < 0.001 versus the control group. After treatment of LNCaP (**c**) and PC-3 (**d**) cells with apigetrin, spheroid formation was observed, measured, and photographed at 48 h to 72 h using a fluorescence microscope and Nikon NIS Elements BR Imaging software. The bar graph represents the percentage of tumor growth. ^#^ *p* < 0.05 (48 h vs. 72 h in control), * *p* < 0.05 and *** *p* < 0.001 versus 48 h control group, and ^$$$^ *p* < 0.001 versus 72 h control.

Next, a cell growth assay was performed to evaluate whether apigetrin inhibits cell growth in long-term (5 days) cell cultures (Figure 2a,b). As shown in Figure 2a,b, apigetrin significantly reduced the growth of LNCaP and PC-3 cells in a dose-dependent manner. LNCaP cells were more susceptible to the cytotoxic effect of apigetrin than PC-3 cells. Apigetrin (50 μM) significantly reduced cell growth by 80.1% and 64% in LNCaP and PC-3 cells, respectively, compared with untreated controls. In addition, we investigated the effect of apigetrin on cell growth using 3D cell culture, which represents the tumor tissue microenvironment. Apigetrin consistently inhibited spheroid growth of LNCaP and PC-3 cells in a time- and dose-dependent manner (Figure 2c,d). LNCaP organoid growth rate increased by 14.3% at 72 h compared with that at 48 h (Figure 2c). PC-3 spheroid growth rate increased by 18.1% at 72 h compared with that at 48 h (Figure 2d). 

### 3.3. High Dose of Apigetrin Induces Apoptosis in LNCaP and PC-3 Cells

**Figure 3:**
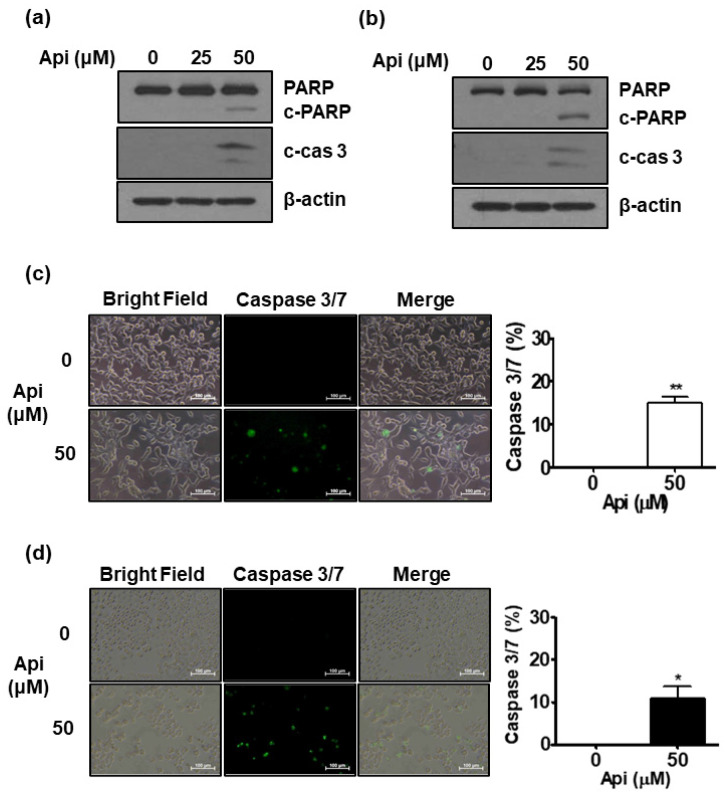
Effect of apigetrin on apoptosis in LNCaP and PC-3 cells. LNCaP (**a**) and PC-3 (**b**) cells were treated with apigetrin (25 and 50 μM) for 48 h. The lysates were subjected to western blotting for PARP, caspase-3, and β-actin. After treatment of LNCaP (**c**) and PC-3 (**d**) cells with apigetrin, the cells were stained with Cell Event^TM^ (caspase-3/7) live dye. Fluorescent apoptotic bodies were counted and expressed as means ± SD. * *p* < 0.05 and ** *p* < 0.01 vs. control.

As shown in Figure 2, 50 μM of apigetrin effectively inhibited cell growth after 5 days of culture. Therefore, we tested whether apigetrin induced apoptosis by measuring the levels of apoptosis-related proteins. As shown in Figure 3a,b, western blotting assay demonstrated that apigetrin increased the cleavage of PARP and caspase-3, attenuated the expression of pro-PARP, and also effectively increased activated caspase-3 (fluorescence dye) to 15% and 10% in LNCaP and PC-3 cells, respectively, compared with the control (Figure 3c,d). 

### 3.4. Apigetrin Decreases AR and HIF-1α Expression in PCa Cells 

**Figure 4:**
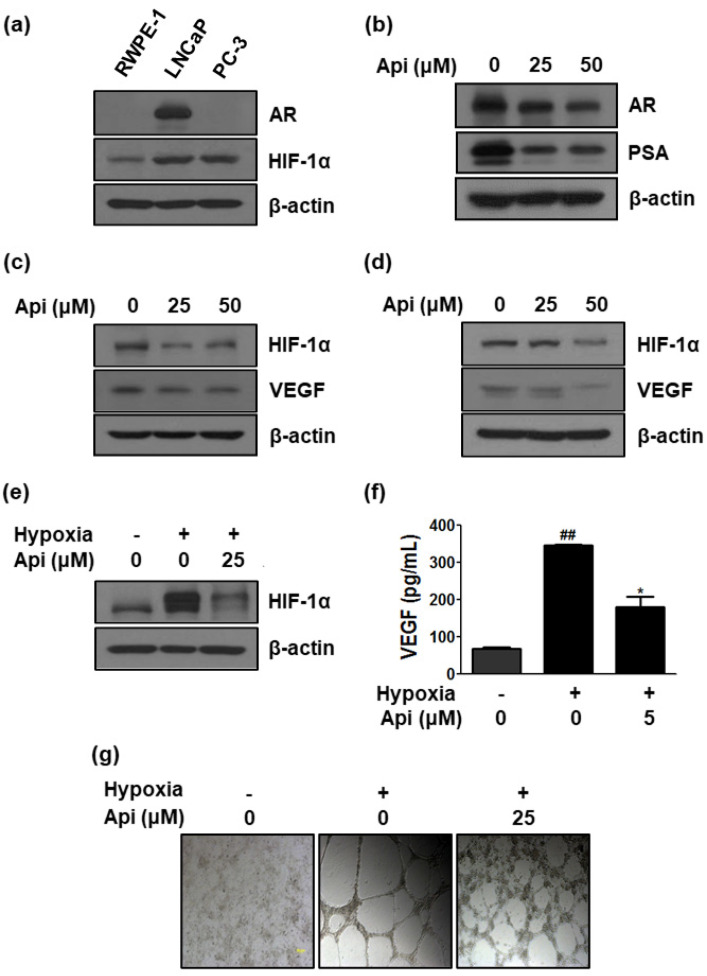
Effect of apigetrin on AR, HIF-1α, and VEGF expression in LNCaP and PC-3 cells and hypoxia-induced angiogenesis in HUVECs. (**a**) Protein expression (basal level) of AR and HIF-1α in prostate cell lines (human normal and cancer cells). LNCaP cells were treated with apigetrin (25 and 50 μM) for 24 h. Cell lysates were prepared and subjected to western blotting to determine the expression of AR and PSA (**b**), HIF-1α, VEGF, and β-actin (**c**). (**d**) PC-3 cells were treated with apigetrin (25 and 50 μM) for 24 h. Cell lysates were prepared and subjected to western blotting to determine the expression of HIF-1α, VEGF, and β-actin. (**e**) LNCaP cells were treated with apigetrin for 24 h under hypoxic conditions. Cell lysates were subjected to western blotting for HIF-1α and β-actin. (**f**) The amount of VEGF in the conditioned medium from LNCaP cells treated with apigetrin and cultured under normoxic or hypoxic conditions for 24 h was measured using an ELISA kit. The bar graph represents the quantification of VEGF level. ^##^ *p* < 0.01 (compared to normal control) and * *p* < 0.05 (compared to hypoxia control). (**g**) Tube formation in HUVECs treated with conditioned medium form hypoxia induced LNCaP cells.

To investigate whether apigetrin regulates HIF-1 α and AR, the basal level of AR and HIF-1 α in prostate cells, including normal (RWPE-1 cells) and cancer cells (LNCaP and PC-3 cells), was examined (Figure 4a). AR was not expressed in PC-3 (AR-independent cells) and RWPE-1 cells but was highly expressed in LNCaP cells. HIF-1α was highly expressed in LNCaP and PC-3 compared with RWPE-1 cells (Figure 4a). Treatment of LNCaP cells with apigetrin (25 and 50 μM) resulted in a decrease in AR and HIF-1α expression. Furthermore, expression of PSA and VEGF that are regulated by AR and HIF-1α, respectively, was reduced by apigetrin (Figure 4b–d).

### 3.5. Hypoxia Upregulates HIF-1α and VEGF Expression and Tube Formation, Wherase Apigetrin Downregulates HIF-1α and VEGF Expression in Hypoxia-Induced LNCaP Cells

LNCaP cells were treated with hypoxia to evaluate whether apigetrin inhibits hypoxia-induced HIF-1α expression (Figure 4e). HIF-1α expression was significantly increased in hypoxia-treated LNCaP cells, but this was reduced by apigetrin (Figure 4e). VEGF level was measured in LNCaP cell culture media to examine the effect of apigetrin on the secretion of VEGF by hypoxia-treated LNCaP cells. VEGF production by hypoxia-induced LNCaP cells increased to 375 pg/mL (almost doubled in its value) compared with the level (65 pg/mL) under normoxic conditions (Figure 4f). In contrast, apigetrin significantly decreased the VEGF secretion (186 pg/mL) compared with the untreated control. In addition, a tube formation assay was carried out with the culture media. As shown in Figure 4g, apigetrin inhibited the capillary tube formation in HUVECs compared with the control group. 

### 3.6. Apigetrin Regulates AKT Expression in LNCaP and PC-3 Cells 

**Figure 5:**
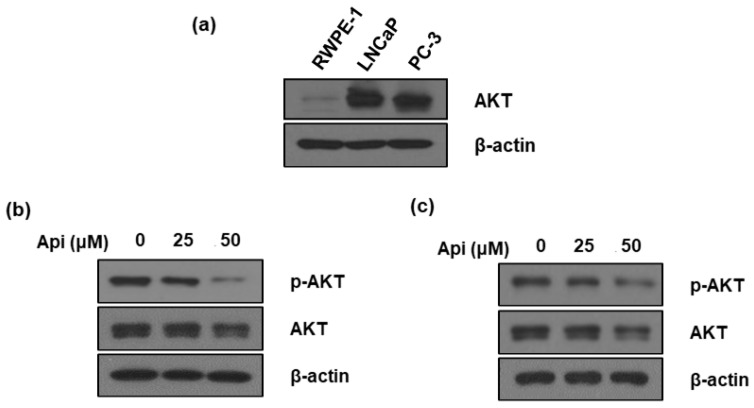
Effect of apigetrin on AKT and p-AKT in LNCaP and PC-3 cells. (**a**) Basal AKT expression in prostate cell lines (human normal and cancer cells). LNCaP cells (**b**) or PC-3 cells (**c**) were treated with apigetrin (25 and 50 μM) for 24 h. Cell lysates were prepared and subjected to western blotting to analyze the expression of AKT, p-AKT, and β-actin.

PI3K/AKT signaling activates and enhances AR and HIF-1α expression and function in PCa [36]. Therefore, we measured the basal level of AKT in prostate cells, including normal and cancer cells. AKT expression was higher in cancer cells (LNCaP and PC-3 cells) compared with that in normal cells (RWPE-1 cells) (Figure 5a). Apigetrin decreased the expression of total and phosphorylated AKT in LNCaP and PC-3 cells (Figure 5b,c). 

### 3.7. AKT Knockout Contributes to the Anti-Cancer Effect of Apigetrin

**Figure 6:**
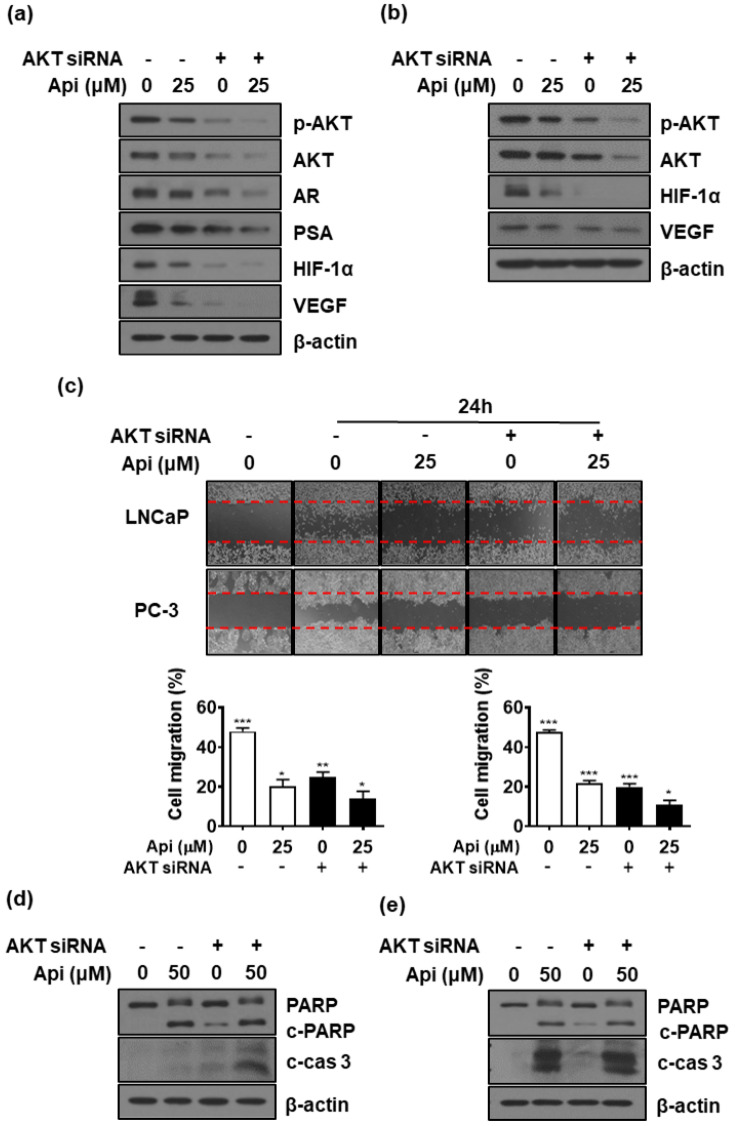
Effect of AKT siRNA in apigetrin-treated LNCaP and PC-3 cells. LNCaP (**a**) and PC-3 cells (**b**) were transfected with AKT siRNA for 48 h and were incubated in the presence or absence of apigetrin for 24 h. Cell lysates were prepared and subjected to western blotting to analyze the expression of AKT, p-AKT, AR, PSA, HIF-1α, VEGF, and β-actin. (**c**) LNCaP and PC-3 cells were transfected with AKT siRNA for 24 h and were incubated in the presence or absence of apigetrin for 24 h. Cell migration was performed using wound-healing assay. Bar graph represents the quantification of cell migration, presented as percentage compared to the control * *p* < 0.05, ** *p* < 0.01, and *** *p* < 0.001. LNCaP (**d**) and PC-3 cells (**e**) were transfected with AKT siRNA for 48 h and were incubated in the presence or absence apigetrin for 48 h. Cell lysates were prepared and subjected to western blotting to determine PARP, cleaved-PARP, cleaved-caspase-3, and β-actin.

As shown in Figure 5b,c, apigetrin downregulated the expression of AKT and phosphorylated AKT. To assess whether the inhibition of AKT affected HIF-1α and AR expression, we examined the regulation of AR and HIF-1α expression using AKT siRNA in LNCaP and PC-3 cells. AKT knockdown decreased the expression of AR and HIF-1α and their downstream proteins PSA and VEGF in PCa cells (Figure 6a,b). AKT siRNA and apigetrin suppressed tumor cell migration of LNCaP and PC-3 cells (Figure 6c). Additionally, AKT siRNA-transfected cells showed a significant increase in PARP and caspase-3 cleavage. Western blot analysis data also revealed that the increased expression of cleaved-PARP and cleaved-caspase-3 induced by apigetrin was further enhanced in AKT-siRNA-transfected cells (Figure 6d,e).

## 4. Discussion

We sought to elucidate the anti-cancer effects and mechanism of action of apigetrin in PCa cell lines that are representative of early- and late-stage PCa.

LNCaP cells are commonly used to study androgen-sensitive early-stage PCa. LNCaP cells are hormone-responsive cells that can be grown in vitro and retain many prostate cell-specific properties [37,38]. Apigetrin decreased AR and PSA expression (Figure 4b) and suppressed migration and long-term growth of LNCaP cells (Figure 1b and Figure 2a). Furthermore, 50 μM of apigetrin induced apoptosis (Figure 3a,c) in these cells. Thus, these data showed that apigetrin inhibits androgen-sensitive early-stage PCa.

LNCaP and PC-3 cells have a homozygous deletion of the PTEN gene and are, therefore, negative for PTEN expression; however, they show high constitutive AKT activity [39,40]. The loss of PTEN expression is an important factor in the progression of metastatic disease in PCa.

PC-3 cells have higher metastatic potential than DU145 cells, which have a moderate metastatic potential, whereas LNCaP cells have relatively low metastatic potential [41]. PC-3 cells do not respond to androgens, glucocorticoids, or fibroblast growth factors [37] and have an aggressive phenotype [38]. Apigetrin suppressed the proliferation and migration in PC-3 cells (Figure 1a,c and Figure 2b,d) and induced apoptosis (Figure 3b,d). Thus, our findings showed that apigetrin exerts anti-cancer activity in early- and advanced-stage PCa.

HIF-1α is an important target for anti-cancer drugs. Many recent studies have demonstrated a strong correlation between elevated levels of HIF-1α and tumor metastasis, angiogenesis, poor patient prognosis, and tumor resistance to therapy [42]. Hypoxia is a typical characteristic of many types of solid tumors [43].

Recent advances have highlighted the critical role of HIF-1α in the development, progression, and metastasis of PCa [44]. Furthermore, a recent meta-analysis study showed that HIF-1α is a potential diagnostic and clinicopathological significance biomarker for PCa [18,45]. The current study demonstrated a novel anti-cancer mechanism of apigetrin via HIF-1α in PCa. Apigetrin was found to suppress the expression of HIF-1α and VEGF, one of its regulated genes, in LNCaP cells and PC-3 cells (Figure 4c–g). Consistent with our data, apigetrin was reported to downregulate HIF-1alpha and VEGF in thyroid cancer [32].

AKT activation is strongly correlated with PCa. The AKT pathway positively regulates protein synthesis, cell cycle, proliferation, invasion, metastasis, angiogenesis, and overall survival in PCa [46,47]. 

Synergistic interactions between AR and AKT in an in vivo prostate regeneration provide evidence that the phosphoinositide-3-kinase (PI3K)/AKT and AR pathways are mechanistically linked [48]. AKT pathway contributes to AR translocation to nuclear and transcriptional activity [49]. The relationship between these factors affects the progression and development of prostate tumor growth. A few studies have demonstrated that AR regulation is downstream of activated AKT; thus, AKT upregulates AR levels in PCa [47].

Additionally, the AKT pathway contributes to HIF-1α accumulation and stabilization in the post-transcriptional and-translational system [50]. Previous studies have shown that HIF-1α is regulated by the PI3K/AKT pathway [51]. Apigetrin inhibited AKT expression in LNCaP and PC-3 cells (Figure 5 and Figure 6).

Collectively, apigetrin exerted anti-proliferative, anti-migratory, and apoptotic effects in LNCaP and PC-3 cells. Apigetrin downregulates AR/PSA and HIF-1α/VEGF signaling by regulating AKT in LNCaP and PC-3 cells. Thus, we provide scientific evidence that apigetrin exerts anti-cancer potential via inhibiting AKT, a key transmitter of HIF-1α and AR signaling in prostate cancer cells.

## 5. Conclusions

In summary, we demonstrated that apigetrin exerted cytotoxicity against LNCaP and PC-3 cells and inhibited cell migration. The long-term 2D and 3D cultures showed that apigetrin enhances cell growth inhibition in long-term cultures. A high dose of apigetrin induced apoptosis as evidenced by increased cleavage of poly ADP-ribose polymerase (PARP) and caspase-3 (c-cas3) in both LNCaP and PC-3 cells. Apigetrin inhibited AR and PSA expression in AR-dependent cell line LNCaP cells. Apigetrin decreased both basal HIF-1α level and the increased HIF-1α level by hypoxia. Apigetrin decreased the enhanced VEGF secretion and HUVECs tube-formation by hypoxia. Silencing of AKT contributed to the anti-cancer activity of apigetrin. In conclusion, apigetrin induces anti-cancer effects by regulating AKT, a key transmitter of HIF-1α and AR signaling in PCa cells.
